# Calibration of a liquid I‐125 source in a syringe

**DOI:** 10.1120/jacmp.v3i3.2565

**Published:** 2002-06-01

**Authors:** Michael T. Gillin

**Affiliations:** ^1^ Department of Radiation Oncology Medical College of Wisconsin 9200 W. Wisconsin Avenue Milwaukee Wisconsin 53226

**Keywords:** Liquid I‐125 source, calibration, radiation therapy

## Abstract

The calibration of a liquid I‐125 source in an older standard dose calibration system is presented. The calibration factor agrees well with factors established by the NIST for newer dose calibration systems. The determination of the source activity is necessary to accurately calculate the time required to deliver the prescribed dose.

PACS number(s): 87.53.Dq, 87.53Jw

RADIATION ONCOLOGY PHYSICS

## INTRODUCTION

A new modality has recently been introduced in the treatment of patients with malignant brain tumors.^1^ This technique involves the surgical placement of a balloon type catheter into a cavity in the patient's brain. The balloon is attached to a flexible catheter shaft, which terminates in an infusion port. The infusion port is placed directly under the skin and is available for the introduction of a liquid radioactive material, which will fill the balloon. Assuming that an appropriate volume of liquid is used, the liquid should assume any shape that the balloon assumes in the cranial cavity. The calculation of dose is based upon tables, provided by catheter vendor. These tables present the dose as a function of treatment prescription, treatment depth, balloon fill volume, maximum transverse balloon diameter, and afterloaded activity. It is the responsibility of the user to determine the afterloaded activity, which is drawn up in a syringe. This paper describes our experience in establishing the calibration factor for the liquid I‐125 sources in our dose calibration system.

## METHODS

Our brachytherapy source calibration system (Capintec Model CRC‐5R) is a well ionization system. Following the recommendations in comprehensive QA for radiation oncology: Report of AAPM Radiation Therapy Committee Task Group 40, we have established a three‐component system, which consists of the radionuclide calibrator, a standard source for the radionuclides which we routinely use, and a long half‐life Cs‐137 reference source, whose calibration is traceable to the NIST^2^ For the solid sources, which we routinely use, namely I‐125 seeds, Cs‐137 tubes, and Ir‐192 seeds, geometry and length dependent factors have been established. The potentiometer of the calibration system has not been adjusted and the calibration factors have been established relative to a single potentiometer setting, 678.

The liquid radiation source, Iotrex™, is a sterile, nonpyrogenic material, which contains sodium 3‐(I125) iodo‐4‐hydroxybenzenesulfonate (I125‐HBS). This material comes to the user in 1 mL unit doses, which contain approximately 7.215 GBq (195 mCi). Depending upon the size of the cavity and thus the size of the balloon, the user must draw the appropriate amount of liquid into a 5 mL syringe and measure the collected activity.^3^

The user is presented with treatment tables, which provide the dwell time as a function of balloon volume, dose to be delivered, and treatment depth. These tables are based upon a “net” afterloaded activity. The net activity is the difference between the total syringe activity assayed prior to the administration of the liquid radioactive material and the residual activity remaining in the dose syringe after the infusion. The residual activity is determined by remeasuring the activity remaining in the syringe, after 1 to 3 mL of saline has been added.

The liquid source presents special challenges in that the material is contained within a plastic syringe, which will attenuate a fraction of the low energy photons emitted in the decay of I‐125. The measurement geometry is fixed in that the syringe can be supported by a plastic insert, which is designed to fit into the ionization well. This insert has a horizontal plate with a central opening, which is located approximately 15 cm above the bottom of the well and which can support the flange portion of the syringe.

The vendor provides two different dial settings for two different dose calibrators. These dial settings are based upon a Report of Test: Experimental Determinations of Capintec Dose Calibrator Correction Factors for I125 Contained in 5 mL Plastic Syringes, by the NIST, December 20, 1999.4 The two dose calibrators (CRC‐12 and CRC‐35R) are more recent models than the one (CRC‐5R) used in this work. Capintec reports that all three of these dose calibrators have essentially similar designs with respect to the ion chamber.

A liquid I‐125 calibration source, which was contained in a 5 mL plastic syringe and whose volume was 2 mL, was obtained.^5^ This source was prepared gravimetrically from a calibrated master solution and is the same solution as is used clinically. The master solution was calibrated in an ion chamber calibrated by the National Physical Laboratory and is directly traceable to national standards. The supplier of this source maintains traceability to the National Institute of Standards and Technology through the Measurements Assurance Programs, as described in US‐NRC Reg. Guide 4.15, Revision 1. The activity of this source is expressed in terms of decays per second. The calibration certificate states that the overall uncertainty at a confidence of 99% is 2.6%. This is based upon a systematic uncertainty of 0.1% and a random uncertainty of 2.5%.

This source is designed to be measured in the syringe. Both the syringe and the needle have been sealed with epoxy. The geometry is the same as the geometry used with the clinical sources.

## RESULTS

The standard initial factors were measured with the dose calibrator set to its normal setting, 678. The readings for zero, background, and other were 00.00 mCi. The voltage was read as 149.9 V. The dial setting was changed to the setting recommended by the vendor and the source in the syringe was placed in the dose calibrator. The measurements are very precise in that the same reading was obtained on the dose calibrator from five independent readings.

The vendor suggested dial setting, 497, produced a reading, which was approximately 4% less than the expected value. The dial setting was then adjusted and the expected reading in milliCuries was obtained. The new dial setting is 475.

## DISCUSSION

A calibration factor, in terms of an appropriate dial setting, has been established for a liquid I‐125 source using an older model of a dose calibrator. This factor is between the two values of the dial setting, as suggested by the NIST, for newer dose calibrators. Table I lists these three settings. It is interesting to observe that the dial setting for the CRC‐5R is between the dial settings for the CRC‐12 and the CRC‐35R. The NIST states that the uncertainties arising from use of these dial settings for the CRC‐12 and CRC‐35R are 1.56% and 1.48%, respectively. The differences in the dial settings may represent slight differences in the individual well chambers studied.

**Table I:** Dial setting for the calibration of a liquid I125 source.

Device	Dial setting recommendation	Source of recommendation
CRC5	475	Measurement
CRC‐12	497	NIST Report
CRC‐35R	469	NIST Report

None of these settings are close to the recommended setting of the manufacturer of the dose calibrator, which is 319. The NIST report states that the use of this setting may produce errors in the activity assay of up to +16% for the 5 mL plastic syringe, depending upon the fill volume.3

A certificate of analysis accompanies each batch of the I‐125 HBS solution, which is to be used clinically. This certificate provides a statement of volume in milliliters and an activity concentration in mCi/ml. The typical uncertainty of the volume delivered is on the order of ±8%. The user draws up some or all of this volume into a syringe.

In actual clinical use, there is activity in both the syringe and the needle. The needle will have different photon attenuation characteristics than the syringe. However, the amount of liquid in an 18 gauge, 1 in. long needle and hub is small, being less than 0.1 mL. This effect is ignored in this work.

## CONCLUSION

It is possible to establish a three component calibration for this liquid I‐125 source. The use of a standard, which is contained in the same type of holder used clinically, eliminates the uncertainties associated with removing radioactive liquid from glass ampoules and estimating the remaining activity. An accurate measurement of the activity to be injected will result in a consistent dose administered to the patient. This approach can be used with other dose calibrators for which there are no recommended dial settings.
